# Racial Disparities in Mortality Rates among Patients with Hepatic Steatosis, Non-Alcoholic Fatty Liver Disease, and Non-Alcoholic Steatohepatitis: Insights from NHANES III Data

**DOI:** 10.1007/s40615-025-02317-9

**Published:** 2025-03-04

**Authors:** Shahrzad Bazargan-Hejazi, Cameron Hines, Myra Usmani, Chris Argueta, Deyu Pan, Arleen F. Brown

**Affiliations:** 1 Department of Psychiatry and Biobehavioral Sciences, College of Medicine, Charles R. Drew University of Medicine and Science and UCLA David Geffen School of Medicine, Los Angeles, CA USA; 2 University of California, Riverside, College of Natural and Agricultural Sciences, Riverside, CA 92507 USA; 3 College of Medicine, Charles R. Drew University of Medicine and Science, Los Angeles, CA USA; 4 Charles R. Drew University of Medicine and Science, Los Angeles, CA USA; 5 UCLA Ronald Reagan Medical Center and Olive View Medical, Los Angeles, CA USA; 6 Department of Medical Education, Alice L. Walton School of Medicine, Bentonville, USA

**Keywords:** NAFLD, NAFL, NASH, Racial disparities, Liver disease, Mortality

## Abstract

**Background:**

Insufficient research has been done on NASH-related cirrhosis mortality and potential racial disparities in mortality rates.

**Objective:**

This study aims to analyze racial differences in mortality rates among patients with non-alcoholic fatty liver disease (NAFLD), non-alcoholic fatty liver (NAFL), and non-alcoholic steatohepatitis (NASH), hypothesizing that hazard ratios for mortality among patients with NAFLD, NAFL, and NASH would be significantly different for Mexican American patients compared to other racial groups.

**Methods:**

Data from NHANES III (1988–1994) representing the U.S. population were analyzed. Bivariate analysis and Cox proportional hazards models were employed to determine mortality rates and predictors across different racial/ethnic groups, adjusting for variables age, gender, smoking status (current, former, non-smoker), BMI (normal, overweight, obese), and a series of biomarkers.

**Results:**

The prevalence of liver diseases in the sample was: NAFLD (12.1%), NAFL (20.0%), and NASH (3.1%). Deceased patients with NASH had the highest weighted mortality rate (50.6%), followed by NAFLD (39.1%) and NAFL (35.5%). Compared to White patients, Black and Mexican American patients exhibited lower mortality rates for NAFLD. Mexican American patients also had lower mortality rates for NFAL and NASH. White patients showed higher hazard ratios (HR) for NAFLD and NAFL compared to Black and Mexican–American patients. However, for NASH, there were no significant differences in HR between racial/ethnic groups.

**Conclusions:**

Despite higher prevalence rates among Mexican American and Black patients, their mortality rates for NAFLD, NAFL, and NASH were comparable or lower than those for Whites. This highlights the need for further research to inform better management and treatment strategies.

## Background

Nonalcoholic fatty liver disease (NAFLD) is a spectrum of liver conditions caused by the accumulation of fat in the liver cells unrelated to significant alcohol consumption. According to the National Institute of Diabetes and Digestive and Kidney Diseases (NIDDK) [1] NAFLD encompasses Nonalcoholic Fatty Liver (NAFL), which is a form of NAFLD characterized by fat buildup in the liver with little to no inflammation or liver damage, and Nonalcoholic Steatohepatitis (NASH), a more severe form of NAFLD that includes liver inflammation and damage in addition to fat accumulation.

If left untreated, NASH can progress to advanced liver conditions such as fibrosis, cirrhosis, and liver failure. [2, 3] Diagnosis, as per NAFLD practice guidelines from the American College of Gastroenterology (ACG) and the World Gastroenterology Organization (WGO), requires evidence of hepatic steatosis, typically confirmed through imaging or histological examination. [4, 5] While liver biopsy remains the gold standard for diagnosis, its invasive nature and cost often lead to the use of non-invasive methods such as ultrasound and metabolic laboratory tests as practical alternatives [4].

Globally, NAFLD is on the rise, with the prevalence estimates ranging from 25 to 45%, and is anticipated to surpass alcohol as the leading cause of cirrhosis and the need for liver transplant. [5, 6] Current data indicates an estimated prevalence of 25–30%, [4] mirroring the prevalence of conditions associated with metabolic syndrome, such as central adiposity, obesity, insulin resistance, metabolic syndrome, and type 2 diabetes. [3, 7, 8] Central adiposity and obesity, especially in morbidly obese individuals, strongly correlate with NAFLD, reaching a prevalence of 80% in this population. [4]The prevalence of NAFLD increases by a factor of 3.5 in obese individuals compared with normal-weight individuals. [9]The escalating global population further contributes to the increasing rates of non-alcoholic steatohepatitis (NASH) and its impact on healthcare. The economic burden is substantial, with the total annual cost of care per NAFLD patient estimated at $7,804 for a new diagnosis and $3,789 for long-term management. [10]

NASH, similar to cirrhosis, is associated with elevated mortality, encompassing acute liver failure and comorbidities. [11] Epidemiological studies reveal race/ethnicity variations in NASH, highlighting disparities in medical care and treatment [12, 13] with Hispanics having the highest rates (58.3%), followed by Whites (44.4%) and African Americans (35.1%). [14–16] Within the Hispanic population, disparities based on country of origin highlight the complex interplay of genetic and environmental factors, and socioeconomic disparities. [17, 18]

While extensive research exists on cirrhosis mortality, insufficient studies have been conducted on NASH-specific causes of cirrhosis and potential racial variations in mortality rates. With NASH's increasing role in causing cirrhosis, [19] there is a critical need to focus on understanding this specific etiology to enhance management and treatment strategies. In the present study we aimed to identify and analyze racial disparities in mortality rates among patients diagnosed with hepatic steatosis, NAFLD, and NASH by using data from the National Health and Nutrition Examination Survey (NHANES III) database. We hypothesized that hazard ratios for mortality among patients with hepatic steatosis, NAFLD, and NASH would be significantly different for Mexican American patients compared to other racial groups, even after adjusting for potential confounders such as age, gender, socioeconomic status, comorbidities, and treatment variables. Identifying the racial patterns of mortality among these patients could benefit future research aimed at identifying potential barriers to the effective management of these conditions.

## Methods

In this observational study, we analyzed data from the National Health and Nutrition Examination Survey III (NHANES III) 1988–1994. NHANES is a cross-sectional survey conducted by the National Center for Health Statistics, using a stratified multistage probability sample to obtain a representative sample of the total civilian, non-institutionalized U.S. population. The CDC had institutional review board approval for NHANES, and informed consent was obtained from all participants. Trained interviewers administered surveys in participants’ homes to ascertain the information collected. In addition, physical examinations and laboratory testing using blood and urine samples were conducted at mobile examination centers.

### Eligibility Criteria

**Fig. 1:**
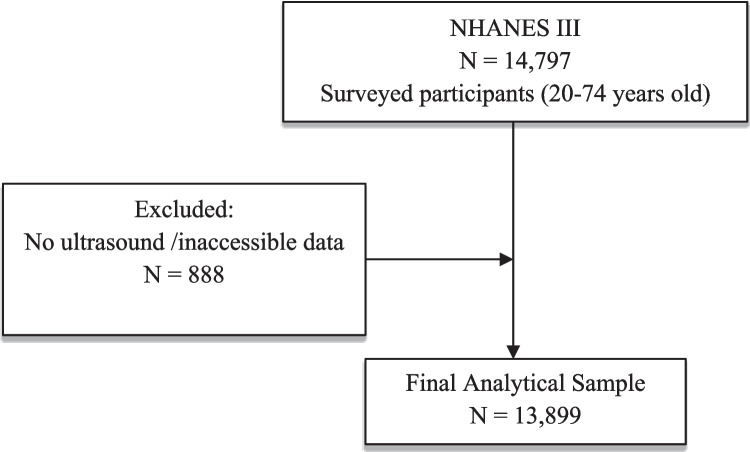
Flowchart of study participants NHANES, National Health and Nutrition Examination Survey

The eligible population comprised participants aged 20 to 74 years (n = 14,797). We excluded individuals with no ultrasound data or inaccessible results (n = 887), resulting in an analytical sample of 13,899 participants. Ultrasound data were used to identify participants with no, mild, moderate, or severe hepatic steatosis (HS). Participants with moderate or severe HS were further evaluated to differentiate between nonalcoholic fatty liver disease (NAFLD) and other causes of liver disease. We excluded individuals with excessive alcohol use, elevated transferrin saturation levels > 50%, or those taking prescription medications known to induce HS (Fig. 1). After these exclusions, participants with moderate or severe HS were classified as having NAFLD. (Fig. 1).

### Study Measures

To distinguish nonalcoholic fatty liver (NAFL) from nonalcoholic steatohepatitis (NASH) within the NAFLD group, we used a combination of clinical and laboratory data. Participants with moderate or severe HS but without evidence of liver inflammation or damage (e.g., elevated liver enzymes such as alanine aminotransferase (ALT) and aspartate aminotransferase (AST) or other markers of hepatic injury were classified as having NAFL. Those with moderate or severe HS accompanied by elevated markers of inflammation and liver damage were categorized as having NASH.

We used four categories to measure race/ethnicity: non-Hispanic White (White), non-Hispanic Black (Black), Mexican–American, and others. Mortality was measured by those who were alive after the 2015 set of NHANES data, and those who were deceased based on Mortality Index information attached to NHANES. Chronic hepatitis B was defined as positive results for both the hepatitis B surface antigen (HBsAg: SAP) and hepatitis B core antibody (anti-HBc: HBP) tests. Chronic hepatitis C was defined as positive results for both the hepatitis C antibody (HCV: HCP) and RNA tests (HCPRNA). Excessive alcohol use was defined as an average of more than 2 drinks/day for men or 1 drink/day for women. Average alcohol use was determined by multiplying the responses to the two questions: “# days drank alcohol in past 12 months” and “average drinks per day on drinking day” and dividing by 365 to get a daily average. In addition to the aforementioned variables, we included a range of demographic and clinical variables, as well as biomarkers, as confounding variables. Specifically, we utilized age (categorized as 20–34, 35–49, 50–64, and 65 +), gender (male, female), smoking status (current, former, non-smoker), BMI (classified as normal, overweight, and obese), and biomarkers such as fasting serum glucose, insulin, lipids, and transferrin saturation (Table 1). Of the NAFLD sample, we identified those with NASH using the HAIR score (measured by hypertension, alanine aminotransferase level, and insulin resistance) [11].

**Table 1:** Characteristics of study sample

Characteristics	Total	Alive	Deceased	*p*-value
n	13899	9627	4272	
Age				< 0.0001
20–34	5019 (37.3%)	4607 (94.2%)	412 (5.8%)	
35–49	3953 (32.3%)	3241 (86.5%)	712 (13.5%)	
50–64	2890 (19.5%)	1474 (56.3%)	1416 (43.7%)	
65 +	2037 (11.0%)	305 (16.2%)	1732 (83.8%)	
Gender				0.0002
Male	6504 (48.5%)	4190 (74.0%)	2314 (26.0%)	
Female	7395 (51.5%)	5437 (77.4%)	1958 (22.6%)	
Race/ethnicity				< 0.0001
White	5064 (75.5%)	3279 (74.9%)	1785 (25.1%)	
Black	4089 (11.0%)	2861 (74.1%)	1228 (25.9%)	
Mexican American	4162 (5.5%)	3019 (79.3%)	1143 (20.7%)	
Other	584 (8.0%)	468 (83.7%)	116 (16.3%)	
NAFLD				< 0.0001
Yes	2051 (12.1%)	1154 (60.9%)	897 (39.1%)	
No	11848 (87.9%)	8473 (77.8%)	3375 (22.2%)	
NAFL				< 0.0001
Yes	3163 (20.0%)	1886 (64.5%)	1277 (35.5%)	
No	10736 (80.0%)	7741 (78.6%)	2995 (21.4%)	
NASH				< 0.0001
Yes	545 (3.1%)	242 (49.4%)	303 (50.6%)	
No	13173 (96.9%)	9276 (76.7%)	3897 (23.3%)	
Smoking status				< 0.0001
Current	3951 (29.8%)	2583 (73.0%)	1368 (27.0%)	
Former	3193 (24.9%)	1830 (66.0%)	1363 (34.0%)	
Non-smoker	6754 (45.3%)	5214 (83.0%)	1540 (17.0%)	
Alcohol use				< 0.0001
Current Drinkers	6599 (56.7%)	4910 (80.7%)	1689 (19.3%)	
Historical drinkers	4727 (31.1%)	2964 (67.7%)	1763 (32.3%)	
Never drinkers	2249 (12.2%)	1535 (73.1%)	714 (26.9%)	
BMI				< 0.0001
Normal	5419 (45.0%)	4012 (80.9%)	1407 (19.1%)	
Overweight	4750 (32.4%)	3219 (74.6%)	1531 (25.4%)	
Obese	3704 (22.6%)	2386 (67.3%)	1318 (32.7%)	
Diabetes				< 0.0001
Yes	1035 (4.9%)	326 (35.8%)	709 (64.2%)	
No	12846 (95.1%)	9290 (77.8%)	3555 (22.2%)	
Hypertension				< 0.0001
Yes	3718 (22.0%)	1544 (47.9%)	2174 (52.1%)	
No	10036 (78.0%)	7968 (83.5%)	2068 (16.5%)	
Fasting serum glucose (mg/dL)	96.10 ± 0.73	92.49 ± 0.65	107.38 ± 1.24	< 0.0001
Normal (< 100 mg/dL)	9617 (77.2%)	7259 (80.4%)	2358 (19.6%)	< 0.0001
Pre-DM (100–125)	2578 (18.0%)	1556 (66.4%)	1022 (33.6%)	
DM (≥ 126 mg/dL)	925 (4.8%)	284 (38.0%)	641 (62.0%)	
Insulin (uU/mL)	10.91 ± 0.28	10.04 ± 0.25	13.65 ± 0.42	< 0.0001
< 25 uU/mL	12264 (94.4%)	8660 (76.9%)	3604 (23.1%)	< 0.0001
≥ 25 uU/mL	1034 (5.6%)	550 (57.7%)	484 (42.3%)	
Serum cholesterol (mg/dL)	203.06 ± 0.80	198.23 ± 0.88	218.19 ± 1.08	
Good(< 200 mg/dL)	6538 (50.1%)	5059 (83.2%)	1479 (16.8%)	< 0.0001
Elevated (200–239)	4167 (31.2%)	2740 (71.4%)	1427 (29.6%)	
High (≥ 240)	2566 (18.7%)	1394 (63.2%)	1172 (36.8%)	< 0.0001
Transferrin saturation (%)	26.54 ± 0.24	26.83 ± 0.27	25.65 ± 0.31	0.0015
< 45%	12529 (93.4%)	8653 (75.3%)	3876 (24.7%)	
≥ 45%	772 (6.6%)	561 (82.3%)	211 (17.7%)	0.0002
Aspartate amino transferase (AST)	21.51 ± 0.18	21.04 ± 0.18	22.99 ± 0.39	< 0.0001
Elevated (> 40 U/L)	654 (3.7%)	376 (63.3%)	278 (36.7%)	< 0.0001
Normal (≤ 40 U/L)	12470 (96.3%)	8725 (76.3%)	3745 (23.7%)	
Alanine amino transferase (ALT)	18.11 ± 0.42	18.22 ± 0.41	17.78 ± 0.55	0.2501
Elevated (> 56U/L)	384 (2.2%)	260 (71.6%)	124 (28.4%)	0.2174
Normal (≤ 56 U/L)	12740 (97.8%)	8841 (75.9%)	3899 (24.1%)	
Hepatitis B virus				0.0001
Yes	1061 (6.1%)	618 (66.6%)	443 (33.4%)	
No	12132 (93.9%)	8527 (76.3%)	3605 (23.7%)	
Hepatitis C virus				0.0342
Yes	351 (2.3%)	200 (66.5%)	151 (33.5%)	
No	12830 (97.7%)	8936 (76.0%)	3894 (24.0%)	

### Statistical Analysis

Descriptive statistics were used to characterize the study sample, presenting mean and standard error (SE) for continuous variables and unweighted numbers and weighted percentages for categorical variables. Bivariate analysis included the Chi-square test for categorical variables, t-test, and ANOVA for continuous variables after satisfying the normality assumptions to determine the statistical difference between the racial/ethnic groups in the prevalence of NASH. Non-parametric tests were used for the independent variables with non-normal distribution data. The Cox proportional hazards model was used to determine the predictors of NAFLD, NAFL, and NASH, adjusting for the confounding variables. We presented the results as an adjusted hazards ratio (HR) and 95% confidence interval (CI), and statistical significance was determined by a p-value of < 0.05. Data were analyzed using SAS (Release V9.4; SAS, Inc). Sample weights provided by the National Center for Health Statistics NCHS were applied to correct for differential selection probabilities and to adjust for non-coverage and non-response. All estimates were appropriately weighted according to NHANES, with due consideration given to the survey design.

## Results

The study sample comprised 13,899 patients, with the ethnic composition detailed in Table 1, column 1. The breakdown included non-Hispanic White patients (75.5%), non-Hispanic Black patients (11.0%), Mexican American patients (5.5%), and Others (8%). Lifestyle variances, comorbidities, and pertinent laboratory values were presented in this table. The prevalence of liver disease in the sample was as follows: NAFLD at 12.1%, NAFL at 20.0%, and NASH at 3.1%. Deceased patients with NASH showed a weighted average of 50.6%, followed by NAFLD (39.1%) and NAFL (35.5%).

Table 2 presents the mortality for NAFLD, NAFL, NASH, and other covariates by race/ethnicity and indicates statistically significant differences between mortality due to these conditions and race when compared with the White group. Mortality rates with NAFLD in White, Black, and Mexican American patients were 44.0%, 35.4%, and 30.1%, respectively. The Black and Mexican American patients had significantly lower mortality rates compared with their White counterparts (p < 0.05). Regarding NAFL, among the White patients, the mortality rate was 38.8% vs. 21.7% without NAFL. In the Black group, the mortality rate was 35.8% with NAFL vs.23.9% without NAFL. For Mexican Americans, it was 25.8% vs. 18.7%. The Mexican American patients had a significantly lower mortality rate compared with the White patients (p < 0.05). With respect to NASH, the mortality rate was 54.1% in the White patients, 53.5%, and 33.8% in the Black and Mexican American patients, respectively. The Mexican American patients had a significantly lower mortality rate compared to their White counterparts (p < 0.05).

**Table 2:** Characteristics of study sample by mortality across race and ethnicity

	White (*n* = 5064)		Black (*n* = 4089)		Mexican American (*n* = 4162)		Other (*n* = 584)	
	Alive (*n* = 3279)	Deceased (*n* = 1785)	Alive (*n* = 2861)	Deceased (*n* = 1228)	Alive (*n* = 3019)	Deceased (*n* = 1143)	Alive (*n* = 468)	Deceased (*n* = 116)
Age								
20–34	1281 (95.0%)	75 (5.0%)	1488 (91.9%)	136 (8.1%)*	1640 (88.8%)	190 (11.2%)*	198 (95.5%)	11 (4.5%)
35–49	1136 (87.4%)	185 (12.6%)	1012 (80.2%)	274 (19.8%)*	934 (81.6%)	235 (18.4%)*	159 (89.3%)	18 (10.7%)
50–64	715 (56.7%)	600 (43.3%)	310 (45.0%)	419 (55.0%)*	358 (56.6%)	349 (43.4%)	91 (65.8%)	48 (34.2%)
65 +	147 (15.5%)	925 (84.5%)	51 (12.8%)	399 (87.2%)	87 (21.2%)	369 (78.8%)	20 (32.7%)	39 (67.3%)*
Gender								
Male	1406 (73.4%)	936 (26.6%)	1174 (70.1%)	663 (29.9%)	1413 (76.7%)	661 (23.3%)	197 (83.2%)	54 (16.9%)*
Female	1873 (76.4%)	849 (23.6%)	1687 (77.3%)	565 (22.7%)	1606 (82.2%)	482 (17.8%)*	271 (84.1%)	62 (15.9%)
NAFLD								
Yes	344 (56.0%)	387 (44.0%)	75 (64.6%)	194 (35.4%)*	469 (69.9%)	300 (30.1%)*	66 (89.8%)	16 (10.2%)*
No	2935 (77.5%)	1398 (22.5%)	2586 (75.3%)	1034 (24.7%)	2550 (81.1%)	843 (18.9%)*	402 (82.7%)	100 (17.3%)
NALF								
Yes	566 (61.2%)	540 (38.8%)	423 (64.2%)	\283 (35.8%)	805 (74.2%)	428 (25.8%)*	92 (85.1%)	26 (14.9%)*
No	2713 (78.3%)	1245 (21.7%)	2438 (76.1%)	945 (23.9%)	2214 (81.3%)	715 (18.7%)	376 (83.3%)	90 (16.7%)
NASH								
Yes	66 (45.9%)	124 (54.1%)	52 (46.5%)	68 (53.5%)	112 (66.2%)	105 (33.8%)*	12 (72.6%)	6 (27.4%)
No	3190 (76.0%)	1637 (24.0%)	2776 (75.0%)	1136 (25.0%)	2857 (80.0%)	1015 (20.0%)*	453 (83.9%)	109 (16.1%)*
Smoking status								
Current	900 (73.2%)	516 (26.8%)	910 (68.0%)	518 (32.0%)*	675 (76.2%)	300 (23.8%)	98 (78.3%)	34 (21.7%)
Former	830 (65.1%)	710 (34.9%)	376 (63.5%)	290 (36.5%)	556 (72.4%)	339 (27.6%)*	68 (78.3%)	24 (21.7%)
Non-smoker	1549 (82.6%)	559 (17.4%)	1575 (81.8%)	419 (18.2%)	1788 (83.1%)	04 (16.9%)	302 (87.2%)	58 (12.8%)
Alcohol use								
Current Drinkers	1911 (80.7%)	741 (19.3%)	1332 (77.8%)	459 (22.2%)	1479 (81.9%)	448 (18.1%)	188 (85.2%)	41 (14.8%)
Historical drinkers	986 (65.8%)	783 (34.2%)	929 (69.4%)	508 (30.6%)	928 (76.0%)	435 (24.0%)*	121 (82.0%)	37 (18.0%)*
Never drinkers	339 (68.0%)	241 (32.0%)	516 (74.5%)	210 (25.5%)	544 (77.9%)	236 (22.1%)*	136 (84.9%)	27 (15.1%)*
BMI								
Normal	1576 (80.7%)	632 (19.3%)	1112 (76.6%)	412 (23.4%)*	1091 (82.1%)	318 (17.9%)	233 (86.2%)	45 (13.8%)*
Overweight	1048 (73.4%)	644 (26.6%)	898 (74.6%)	397 (25.4%)	1137 (79.5%)	453 (20.5%)*	136 (82.1%)	37 (17.9%)
Obese	655 (64.7%)	509 (35.3%)	851 (70.6%)	414 (29.4%)*	782 (75.5%)	361 (24.5%)*	98 (79.5%)	34 (20.5%)
Diabetes								
Yes	68 (33.2%)	221 (66.8%)	99 (34.0%)	222 (66.0%)	140 (47.2%)	251 (52.8%)*	19 (56.4%)	15 (43.6%)
No	3206 (76.9%)	1561 (23.1%)	2762 (77.2%)	1003 (22.8%)	2873 (81.5%)	890 (18.5%)*	449 (84.8%)	101 (15.2%)*
Hypertension								
Yes	539 (46.2%)	912 (53.8%)	571 (49.3%)	733 (50.7%)	364 (54.4%)	481 (45.6%)*	70 (61.8%)	48 (38.2%)
No	2730 (82.9%)	866 (17.1%)	2277 (84.4%)	490 (15.6%)	2574 (83.7%)	645 (16.3%)	387 (87.9%)	67 (12.1%)
Fasting serum glucose (mg/dL)	91.87 ± 0.81	106.34 ± 1.43	94.07 ± 0.96	113.62 ± 2.34	94.23 ± 0.64	113.50 ± 2.45	94.79 ± 1.28	104.41 ± 4.00
Normal (< 100)	2598 (79.8%)	1050 (20.2%)	2069 (78.7%)	662 (21.3%)	2266 (83.2%)	588 (16.8%)*	326 (86.6%)	58 (13.4%)*
Pre-DM (100–125)	488 (64.1%)	450 (35.9%)	460 (70.0%)	250 (30.0%)	512 (74.9%)	287 (25.1%)*	96 (75.2%)	35 (24.8%)
DM (≥ 126 mg/dL)	67 (33.6%)	208 (66.4%)	91 (36.1%)	200 (63.9%)	105 (41.8%)	217 (58.2%)	21 (75.0%)	16 (25.0%)*
Insulin (uU/mL)	9.31 ± 0.31	13.07 ± 0.48	12.52 ± 0.36	16.57 ± 0.98	12.29 ± 0.38	15.12 ± 0.73	11.92 ± 0.68	14.86 ± 1.21
< 25 uU/mL	3087 (76.1%)	1579 (23.9%)	2452 (75.5%)	973 (24.5%)	2709 (80.1%)	959 (19.9%)*	412 (84.3%)	93 (15.7%)*
≥ 25 uU/mL	102 (51.8%)	153 (48.2%)	215 (61.1%)	162 (38.9%)	196 (68.4%)	152 (31.6%)*	37 (76.7%)	17 (23.3%)*
Lipids (mg/dL)								
Serum cholesterol (mg/dL)	199.23 ± 1.08	219.82 ± 1.26	194.15 ± 0.76	212.97 ± 1.77	195.50 ± 1.20	210.15 ± 2.62	196.16 ± 2.91	212.09 ± 4.73
Good (< 200 mg/dL)	1629 (83.2%)	542 (16.8%)	1544(79.6%)	468 (20.4%)*	1628 (83.5%)	426 (16.5%)	258 (88.2%)	43 (11.8%)
Elevated (200–239)	986 (70.1%)	658 (29.9%)	766 (73.6%)	351 (26.4%)	877 (77.9%)	382 (22.1%)*	111 (78.5%)	36 (21.5%)
High (≥ 240)	570 (62.3%)	533 (37.7%)	339 (56.7%)	310 (43.3%)	405 (66.5%)	299 (33.5%)	80 (77.7%)	30 (22.3%)*
Transferrin saturation (%)	27.39 ± 0.36	25.76 ± 0.31	24.04 ± 0.25	23.91 ± 0.36	25.87 ± 0.30	25.75 ± 0.44	25.86 ± 0.56	27.64 ± 1.24
< 45%	2954 (74.3%)	1645 (25.7%)	2559 (74.2%)	1093 (25.8%)	2717 (79.3%)	1034 (20.7%)*	423 (83.8%)	104 (16.2%)*
≥ 45%	235 (83.2%)	86 (16.8%)	109 (75.4%)	42 (24.6%)	191 (77.7%)	78 (22.3%)	26 (83.2%)	5 (16.8%)
Aspartate amino transferase (AST)	20.60 ± 0.22	22.07 ± 0.37	21.67 ± 0.39	25.88 ± 0.96	24.15 ± 0.52	28.66 ± 1.10	22.10 ± 0.68	25.40 ± 1.42
Elevated (> 40 U/L)	68 (63.2%)	60 (36.8%)	104 (52.0%)	101 (48.0%)	184 (70.7%)	107 (29.3%)	20 (71.3%)	10 (28.7%)
Normal (≤ 40 U/L)	3086 (75.3%)	1648 (24.7%)	2516 (75.4%)	1012 (24.6%)	2700 (80.0%)	986 (20.0%)*	423 (84.3%)	99 (15.7%)*
Alanine amino transferase (ALT)	17.72 ± 0.49	17.23 ± 0.59	16.45 ± 0.36	17.51 ± 0.77	23.94 ± 0.66	25.38 ± 0.94	20.86 ± 1.13	19.85 ± 1.66
Elevated (> 56 U/L)	42 (69.2%)	24 (30.8%)	50 (63.8%)	29 (36.2%)	154 (72.4%)	66 (27.6%)	14 (87.9%)	5 (12.1%)
Normal (≤ 56 U/L)	3112 (75.1%)	1684 (24.9%)	2570 (74.4%)	1084 (25.6%)	2730(79.7%)	1027 (20.3%)*	429 (83.4%)	104 (16.6%)*
Hepatitis B virus								
Yes	98 (63.3%)	88 (36.7%)	306 (62.5%)	236 (37.5%)	122 (69.3%)	87 (30.7%)	92 (75.4%)	32 (24.6%)
No	3069 (75.3%)	1634 (24.7%)	2337 (76.2%)	887 (23.8%)	2769 (79.9%)	1007 (20.1%)*	352 (85.5%)	77 (14.5%)*
Hepatitis C virus								
Yes	49 (72.6%)	28 (27.4%)	80 (53.3%)	68 (46.7%)*	59 (59.8%)	46 (40.2%)	12 (60.6%)	9 (39.4%)
No	3119 (74.9%)	1695 (25.1%)	2558 (75.1%)	1052 (24.9%)	2827 (79.9%)	1047 (20.1%)*	432 (84.3%)	100 (15.7%)*
Hepatitis B&C virus								
Yes	132 (65.6%)	108 (34.4%)	354 (62.2%)	264 (37.8%)	161 (65.5%)	121 (34.5%)	95 (74.1%)	36 (25.9%)
No	3036 (75.4%)	1615 (24.6%)	2289 (76.6%)	759 (23.4%)	2731 (80.4%)	973 (19.6%)*	349 (86.0%)	73 (14.0%)*

^*^p < 0.05 comparing with Whit

### Cox Progression Analysis

Table 3, 4 and 5 utilized Cox progression analysis to establish hazard ratios for NAFLD, NAFL, and NASH while controlling for confounding variables/comorbidities. Mexican American patients demonstrated lower hazard ratios (HR) for all three conditions, although some findings were insignificant. Regarding NAFLD vs. no NAFLD: White patients showed an HR of 1.29 (95% CI 1.14–1.48) (p = 0.0003), Black patients HR = 1.16 (95% CI 0.96–1.40) (p = 0.1218), and Mexican American HR = 1.21 (1.04–1.40) (p = 0.0139). For NFAL vs no NAFL: White patients demonstrated an HR = 1.21 (95% CI 1.06–1.39) (p = 0.0050), Black patients HR = 1.22 (95% CI 0.97–1.54) (p = 0.0829), and Mexican Americans: HR = 1.02 (95% CI 0.84–1.24) (p = 0.8501). The hazard ratio for the “Other” group, primarily Asian Americans, could not be calculated due to insufficient sample size. For NASH vs. no NASH: White patients had an HR of 1.16 (95% CI 0.86–1.57) (p = 0.3295), Black patients HR = 1.20 (95% CI 0.94–1.53) (P = 0.1329), and Mexican–American patients HR = 0.84 (95% CI 0.57–1.22) (p = 0.3457).

**Table 3:** Hazard Ratio of Mortality for NAFLD by Race and Ethnicity (mortality data updated to 2015)

	White		Black		Mexican American	
	HR (95%CI)	*p*-value	HR (95%CI)	*p*-value	HR (95%CI)	*p*-value
NAFLD						
Yes	1.29 (1.14–1.48)	0.0003	1.16 (0.96–1.40)	0.1218	1.21 (1.04–1.40)	0.0139
No	Ref		Ref		Ref	
Age						
20–34	Ref		Ref		Ref	
35–49	2.54 (1.95–3.32)	< 0.0001	2.10 (1.65–2.69)	< 0.0001	1.66 (1.29–2.14)	0.0003
50–74	13.73 (10.59–17.81)	< 0.0001	8.24 (6.56–10.36)	< 0.0001	4.79 (3.79–6.04)	< 0.0001
Gender						
Male	1.21 (1.05–1.38)	0.0077	1.56 (1.41–1.73)	< 0.0001	1.51 (1.26–1.82)	< 0.0001
Female	Ref		Ref		Ref	
Smoking status						
Current	2.47 (2.17–2.82)	< 0.0001	1.66 (1.38–2.00)	< 0.0001	1.43 (1.19–1.71)	0.0003
Former	1.46 (1.27–1.68)	< 0.0001	1.10 (0.91–1.32)	0.3106	1.17 (0.94–1.45)	0.1590
Non-smoker	Ref		Ref		Ref	
BMI						
Normal	Ref		Ref		Ref	
Overweight	0.92 (0.77–1.10)	0.3390	0.74 (0.61–0.91)	0.0054	0.81 (0.68–0.96)	0.0152
Obese	1.11 (0.95–1.29)	0.1565	0.85 (0.72–1.00)	0.0469	0.86 (0.73–1.00)	0.0528
Diabetes						
Yes	2.37 (1.96–2.86)	< 0.0001	2.40 (1.99–2.90)	< 0.0001	2.06 (1.66–2.55)	< 0.0001
No	Ref		Ref		Ref	
Hypertension						
Yes	1.70 (1.45–1.99)	< 0.0001	1.89 (1.62–2.21)	< 0.0001	1.58 (1.28–1.95)	< 0.0001
No	Ref		Ref		Ref	
Aspartate amino	1.02 (1.01–1.03)	< 0.0001	1.01 (1.01–1.01)	< 0.0001	1.01 (1.00–1.01)	< 0.0001
Alanine amino	0.98 (0.97–0.99)	0.0005	0.99 (0.99–1.00)	0.076	0.99 (0.99–1.00)	0.0296
Hepatitis B&C virus						
Yes	1.55 (1.11–2.16)	0.0109	1.38 (1.17–1.63)	0.0003	1.23 (0.95–1.58)	0.1137
No	Ref		Ref		Ref

**Table 4:** Hazard ratio of mortality for NAFL by race and ethnicity (mortality data updated to 2015)

	White		Black		Mexican American	
	HR (95%CI)	*p*-value	HR (95%CI)	*p*-value	HR (95%CI)	*p*-value
NAFL						
Yes	1.21 (1.06–1.39)	0.0050	1.22 (0.97–1.54)	0.0829	1.02 (0.84–1.24)	0.8501
No	Ref		Ref		Ref	
Age						
20–34	Ref		Ref		Ref	
35–49	2.56 (1.96–3.34)	< 0.0001	1.96 (1.48–2.59)	< 0.0001	1.68 (1.30–2.16)	0.0002
50–74	13.84 (10.67–17.96)	< 0.0001	8.37 (6.50–10.78)	< 0.0001	4.89 (3.85–6.21)	< 0.0001
Gender						
Male	1.19 (1.04–1.36)	0.0125	1.50 (1.35–1.66)	< 0.0001	1.50 (1.24–1.81)	< 0.0001
Female	Ref		Ref		Ref	
Smoking status						
Current	2.44 (2.14–2.77)	< 0.0001	1.56 (1.30–1.88)	< 0.0001	1.42 (1.18–1.70)	0.0005
Former	1.44 (1.26–1.66)	< 0.0001	0.98 (0.78–1.22)	0.8189	1.16 (0.93–1.44)	0.1882
Non-smoker	Ref		Ref		Ref	
BMI						
Normal	Ref		Ref		Ref	
Overweight	0.92 (0.76–1.10)	0.3287	0.80 (0.65–0.98)	0.0356	0.82 (0.69–0.97)	0.0209
Obese	1.11 (0.96–1.29)	0.1544	0.87 (0.72–1.06)	0.1655	0.88 (0.76–1.03)	0.1139
Diabetes						
Yes	2.41 (2.00–2.91)		2.47 (1.99–3.06)		2.13 (1.69–2.67)	< 0.0001
No	Ref	< 0.0001	Ref	< 0.0001	Ref	
Hypertension						
Yes	1.69 (1.44–1.98)		1.70 (1.42–2.04)		1.58 (1.29–1.94)	< 0.0001
No	Ref	< 0.0001	Ref	< 0.0001	Ref	
Aspartate amino transferase (AST)	1.02 (1.01–1.03)	< 0.0001	1.02 (1.01–1.02)	0.0001	1.01 (1.00–1.02)	< 0.0001
Alanine amino transferase (ALT)	0.98 (0.97–0.99)	0.0004	0.99 (0.98–1.00)	0.0441	0.99 (0.99–1.00)	0.0442
Hepatitis B&C virus						
Yes	1.55 (1.11–2.16)	0.0105	1.40 (1.17–1.66)	0.0004	1.22 (0.94–1.59)	0.1285
No	Ref		Ref

**Table 5:** Hazard ratio of mortality for NASH by race and ethnicity (mortality data updated to 2015)

	White		Black		Mexican American	
	HR (95%CI)	*p*-value	HR (95%CI)	*p*-value	HR (95%CI)	*p*-value
NASH						
Yes	1.16 (0.86–1.57)	0.3295	1.20 (0.94–1.53)	0.1329	0.84 (0.57–1.22)	0.3457
Normal	Ref		Ref		Ref	
Age						
20–34	Ref		Ref		Ref	
35–49	2.57 (1.90–3.49)	< 0.0001	1.96 (1.48–2.59)	< 0.0001	1.75 (1.32–2.31)	0.0003
50–74	13.48 (10.35–17.57)	< 0.0001	8.37 (6.50–10.77)	< 0.0001	5.32 (4.05–7.00)	< 0.0001
Gender						
Male	1.23 (1.05–1.45)	0.0133	1.50 (1.35–1.66)	< 0.0001	1.62 (1.30–2.01)	< 0.0001
Female	Ref		Ref		Ref	
Smoking status						
Current	2.37 (2.00–2.81)	< 0.0001	1.56 (1.30–1.88)	< 0.0001	1.32 (1.07–1.63)	0.0112
Former	1.43 (1.21–1.68)	< 0.0001	0.98 (0.78–1.22)	0.8238	1.06 (0.77–1.46)	0.6224
Non-smoker	Ref		Ref		Ref	
BMI						
Normal	Ref		Ref		Ref	
Overweight	0.87 (0.71–1.08)	0.1962	0.80 (0.65–0.98)	0.0355	0.87 (0.72–1.04)	0.1225
Obese	1.14 (0.94–1.38)	0.1675	0.87 (0.72–1.06)	0.1682	0.85 (0.70–1.04)	0.1190
Diabetes						
Yes	2.27 (1.83–2.83)	< 0.0001	2.47 (1.99–3.07)	< 0.0001	2.28 (1.58–3.28)	< 0.0001
No	Ref		Ref		Ref	
Hypertension						
Yes	1.82 (1.54–2.15)	< 0.0001	1.70 (1.42–2.04)	< 0.0001	1.65 (1.32–2.05)	< 0.0001
No	Ref		Ref		Ref	
Aspartate amino	1.02 (1.01–1.03)	0.0002	1.02 (1.01–1.02)	0.0001	1.01 (1.00–1.01)	< 0.0001
Alanine amino	0.98 (0.97–0.99)	0.0027	0.99 (0.98–1.00)	0.0445	*	
Hepatitis B&C virus						
Yes	1.47 (1.03–2.09)	0.0333	1.40 (1.17–1.66)	0.0004	1.24 (0.92–1.67)	0.1485
No	Ref		Ref			

^*^If Alanine amino is included in the model, the model is unstable because of small sample in some strata

## Discussion

In our study, we aimed to investigate racial disparities in mortality in those with conditions under the NAFLD umbrella. Contrary to our hypothesis, we found that Mexican Americans have a higher prevalence of NAFLD, NAFL, and NASH, as indicated in the literature. [16] However, their mortality rates were not significantly higher compared to their White counterparts. This paradoxical finding is consistent with the “Hispanic paradox,” where Hispanic populations have better or similar health outcomes despite higher disease prevalence. [20] It highlights the suggested interplay of genetic, environmental, and healthcare factors in determining health outcomes across different racial and ethnic groups. [17, 18] Nevertheless, the racial patterns of mortality observed in our study provide a baseline for further investigation into the management of NAFLD, NAFL, and NASH among patients with different racial/ethnic backgrounds.

Our data also suggest that demographic variables such as age and gender are significant factors in the clinical presentation and outcomes of liver diseases. These demographic factors, along with lifestyle and biological markers identified as independent predictors of mortality (as shown in Table 3, 4 and 5), may further indicate underlying disparities in disease management and healthcare access. They suggest that healthcare providers should be aware of these differences and consider them when diagnosing and managing NAFLD, NAFL, and NASH in patients from diverse racial backgrounds.

Our findings align with previous studies highlighting ethnic and racial disparities in NAFLD prevalence and outcomes. A recent study by Abu-Freha et al. (2024) examined the prevalence and management of NAFLD in the Arab population in Israel, emphasizing the interplay of genetic predisposition, cultural dietary practices, and limited healthcare access in shaping NAFLD outcomes. [21] Similar to our findings, the study underscored the need for targeted interventions addressing socioeconomic and cultural determinants of health.

These comparisons reinforce the importance of considering both genetic and environmental factors when examining racial disparities in NAFLD and related conditions. They also highlight the global relevance of our findings, as disparities in NAFLD outcomes extend beyond the USA to other regions with diverse populations.

Additionally, it is important to note the recent adoption of the term metabolic dysfunction-associated steatotic liver disease (MASLD) [22, 23] which reflects an evolving understanding of fatty liver disease. MASLD shifts the focus to metabolic dysfunction as a central criterion, moving away from exclusion-based diagnoses. While this study utilizes NAFLD terminology consistent with the time of data collection (1988–1994), MASLD offers a more inclusive and clinically relevant framework for future research and management. Nevertheless, our findings underscore the importance of investigating the interplay between metabolic health and liver disease outcomes in diverse populations to better inform prevention and treatment strategies.

While this study provides important insights into racial disparities in mortality within the spectrum of NAFLD, there are some limitations to consider. First, we have retained the use of the term NAFLD throughout the manuscript to maintain consistency with the historical context of the NHANES III dataset (1988–1994), which predates the adoption of the term metabolic dysfunction-associated MASLD. Consequently, our findings are framed using the diagnostic criteria and terminology that were standard during the time of data collection. Future research using updated datasets and MASLD criteria could provide a more comprehensive understanding of fatty liver disease in the context of evolving diagnostic standards. Second, the NHANES dataset, with all its richness in providing a plethora of information regarding this study population, primarily focuses on Mexican Americans, who exhibit the highest rates of NAFLD. This limits our ability and certainty to associate differences in mortality with ethnoracial disparities. Considering that globally, Asian Americans have increasingly high rates of NAFLD and NASH, which contradicts their relatively low BMI, efforts to enhance health data collection for this population are needed. [24]. Finally, our study is limited by the absence of detailed information regarding patients, access to care, and overall management of their chronic comorbidities. Existing research on health disparities highlights a lack of access to routine medical care as a significant contributing factor to disparate outcomes among different ethnic groups. [25]

## Conclusions

In conclusion, our study sheds light on patterns of racial disparities in mortality within the spectrum of NAFLD. Despite the higher prevalence of NAFLD, NFAL, and NASH among Mexican Americans and Black populations, their mortality rates were either comparable or lower than those observed in White patients. This finding contributes to a growing body of evidence highlighting the complex interplay between genetic, environmental, and healthcare-related factors with health outcomes across racial and ethnic groups. Additionally, demographic variables such as age, gender, and lifestyle factors were significant predictors of mortality, highlighting underlying disparities in disease management and healthcare access.

Our findings underscore the urgency of prioritizing research to improve prevention, management strategies, and treatment protocols. Consistent guidance and management of NAFLD, NAFL, and NASH through primary care are critical. This could include incorporating patient-centered care, conducting regular screenings for high-risk populations, and addressing barriers to healthcare access such as cost, geographic availability, language, and cultural competence.

Finally, the recent adoption of the term MASLD represents an important shift in understanding fatty liver disease, emphasizing metabolic dysfunction as a key diagnostic feature. While this study retains the term NAFLD to align with the historical context of the NHANES III dataset (1988–1994), MASLD offers a more inclusive framework for future research and clinical practice. This paradigm shift underscores the need to integrate metabolic health into diagnosis and management strategies to better serve diverse patient populations and address disparities in outcomes.

## Data Availability

The data analyzed in this study were obtained from the National Health and Nutrition Examination Survey III (NHANES III) database, conducted between 1988 and 1994. NHANES III data are publicly accessible and can be retrieved through the Centers for Disease Control and Prevention (CDC) website (https://www.cdc.gov/nchs/nhanes/index.htm).
